# Relatively independent inhibitory control and cognitive flexibility deficits on the WCST in children with ADHD: age-related change patterns and cognitive profiles

**DOI:** 10.3389/fpsyt.2026.1874399

**Published:** 2026-07-29

**Authors:** Yixiao Pan, Xuanru Wu, Zhang Lin, FeiFei Zhao

**Affiliations:** Department of Psychological Assessment, Wenzhou Seventh People’s Hospital, Wenzhou, Zhejiang, China

**Keywords:** ADHD, cognitive profiles, cognitive flexibility, executive function, inhibitory control, Wisconsin card sorting test

## Abstract

**Background:**

Debate persists regarding whether executive dysfunction in ADHD arises from a single core deficit or reflects impairments across multiple relatively independent components. Using the Wisconsin Card Sorting Test (WCST), this study examined the relative independence of inhibitory control and cognitive flexibility deficits, their age-related change patterns, and identifiable cognitive profiles in children with ADHD.

**Methods:**

A total of 281 children with ADHD (aged 7–12 years) completed the WCST. Trial-level generalized linear mixed models examined predictors of response accuracy, and Gaussian mixture modeling identified cognitive profiles based on perseverative error rate (PER; inhibitory control) and shape rule disadvantage (SRD; cognitive flexibility).

**Results:**

PER and SRD showed a weak positive correlation (*r* = .245, shared variance = 6.0%). Shape rule exerted a significant main effect on accuracy after controlling for covariates. A significant negative cross-sectional association with age was found for PER but not for SRD, although the age × deficit type interaction was not significant (*p* = .794). Two cognitive profiles were identified: a mild deficit profile (38.1%) with relatively preserved cognitive flexibility, and a dual deficit profile (61.9%) with concurrent impairments.

**Conclusions:**

Inhibitory control and cognitive flexibility deficits are relatively independent in children with ADHD, broadly consistent with multi-component frameworks of executive function. The identification of two cognitive profiles with distinct impairment patterns provides a foundation for comprehensive assessment, though these profiles require further validation through longitudinal and independent cohort studies.

## 1 Introduction

Attention-Deficit/Hyperactivity Disorder (ADHD) is one of the most common neurodevelopmental disorders in childhood, with a pooled global prevalence of approximately 3.0% (95% CI 2.6%–4.5%) (1). Characterized by core symptoms of inattention, hyperactivity, and impulsivity, ADHD is associated with reduced academic achievement, impaired social adaptation, and persistent adverse impacts on occupational functioning and mental health throughout adolescence and adulthood (2).

A substantial body of research has established executive dysfunction as a core cognitive pathophysiological feature of ADHD (3). However, fundamental debate persists regarding the underlying structure and mechanisms of these executive deficits—specifically, whether they arise from a single core impairment or reflect concurrent impairments across multiple relatively independent cognitive components. On one side of this debate, Barkley’s (1997) inhibitory control model posits that impaired behavioral inhibition constitutes the primary deficit in ADHD, with difficulties in working memory, emotional regulation, and other executive functions viewed as secondary consequences (3). This single-core-deficit account has received partial empirical support: children with ADHD show significantly elevated perseverative errors on the Wisconsin Card Sorting Test (WCST)—a classic measure of executive function—with a meta-analysis reporting a weighted mean effect size of 0.46 (95% CI 0.37–0.55) for this index (4). Such errors are interpreted as reflecting an impaired ability to suppress irrelevant or outdated response tendencies. Subsequent meta-analyses of functional magnetic resonance imaging (fMRI) studies have further confirmed reduced activation in regions associated with the ventrolateral prefrontal cortex during inhibitory control tasks in individuals with ADHD, providing critical neuroanatomical evidence for this framework (5).

On the other side of the debate, Miyake et al.’s (2000) unity-diversity model fundamentally challenges the single-core-deficit account. Using latent variable analysis, this model demonstrated that inhibitory control, cognitive flexibility (also known as set-shifting), and working memory updating are three relatively independent components, showing only moderate inter-correlations (r = 0.42–0.63) and contributing differentially to complex executive tasks (6). According to this multi-component perspective, executive function deficits in ADHD need not stem from a single source. Empirical support for this view comes from studies using task-switching paradigms, which have identified specific cognitive flexibility impairments in children with ADHD—characterized by significantly increased switch costs—that are ameliorated by medication (7). Further evidence from the team of Professor Yufeng Wang revealed multi-dimensional working memory deficits in children with ADHD, with strong associations between laboratory-based and ecological indicators (8). More recently, the dual-pathway model of cool/hot executive functions has refined this multi-component perspective, proposing that “cool” executive functions (inhibitory control, working memory, cognitive flexibility) primarily underlie inattentive symptoms, whereas “hot” executive functions (delay discounting, reward processing, emotional regulation) mainly drive hyperactive/impulsive symptoms (9). Thus, the divergence between these frameworks points to a critical unresolved question: Is executive dysfunction in ADHD best characterized as a single core deficit, or as concurrent impairments across multiple relatively independent cognitive components?

The Wisconsin Card Sorting Test (WCST) provides a useful tool for addressing this theoretical controversy. First described by Berg (10) and later standardized by Heaton et al. (11), the WCST has become a classic paradigm for assessing executive function (10, 11). Its key strength lies in its ability to simultaneously and simultaneously capture multiple critical cognitive components—including cognitive flexibility (set-shifting), inhibitory control (impulse regulation), and inductive reasoning—within an integrated task framework. This “one-test, multi-component” characteristic makes it highly suitable for investigating the heterogeneous nature of executive dysfunction in ADHD. Specifically, the WCST enables researchers to directly measure inhibitory control deficits through perseverative errors, precisely quantify cognitive flexibility via rule-switching costs, and capture attentional maintenance and cognitive resource allocation efficiency through dynamic changes in performance over time (12, 13).

Despite these strengths, existing research has not fully exploited the potential of the WCST, and remains limited by several interrelated critical shortcomings. First, the analytical perspective lacks integration. Although most studies report multiple traditional WCST indices (e.g., perseverative errors, number of categories completed), these measures are often treated as independent outcomes, without systematic examination of rule-specific effects, dynamic performance trajectories across task blocks, or relationships between different error types within a unified analytical framework (12, 13). This limits the ability to determine whether deficits in different cognitive components co-occur or vary independently—a distinction central to discriminating between single core deficit and multi-component models. Second, inconsistencies in terminology and scoring systems hinder cross-study comparability. The terms “perseverative responses” and “perseverative errors” are frequently used interchangeably; the scoring systems of Grant & Berg (10) and Heaton et al. (11) are used in parallel, and some studies further modify task rules (10, 11).As a result, findings are difficult to compare directly or to synthesize through meta-analysis. Third, investigation of age effects remains underdeveloped. Although some studies include a range of ages, fine-grained empirical evidence is lacking regarding the developmental patterns of executive dysfunction—especially whether distinct components such as inhibitory control and cognitive flexibility follow differential developmental trajectories with age (14, 15). Critically, potential divergences in developmental trajectories across cognitive components—an issue central to understanding cognitive prognosis in ADHD—have not been adequately addressed. Finally, and with the greatest translational potential, cognitive-based clinical subtyping remains severely understudied. Although heterogeneity in ADHD is widely acknowledged, studies using data-driven approaches to identify ADHD subtypes with distinct cognitive deficit profiles based on the multi-dimensional measurement advantages of the WCST remain scarce (16, 17). More recently, person-centered approaches have further highlighted the multidimensional nature of executive function heterogeneity in ADHD. A comprehensive multi-task assessment found that 89% of children with ADHD demonstrated impairment on at least one executive function, with 54% impaired on a single function and 35% impaired on two or all three functions (18), underscoring the importance of multi-component frameworks for understanding cognitive heterogeneity in ADHD. This has left the considerable potential of the WCST for supporting precise assessment and classification largely untapped, and has prevented cognitive assessment results from being effectively translated into guidance for clinical intervention.

To address these limitations, the present study adopted a trial-level fine-grained analytical approach, strictly adhered to standardized terminology and scoring protocols, and used advanced statistical methods—including generalized linear mixed models (GLMM) and Gaussian mixture modeling (GMM)—to systematically examine WCST performance in children with ADHD. This approach enabled us to investigate three sequential core research questions. First, among children with ADHD, do inhibitory control deficits (indexed by perseverative errors) and cognitive flexibility deficits (indexed by rule-switching difficulties, particularly disadvantages under shape rules) represent mutually independent cognitive impairments? Second, within the critical developmental period of 7–12 years, what age-related change patterns do these two deficits exhibit? Do cognitive flexibility deficits demonstrate greater developmental stability (i.e., less improvement with age)? Third, can data-driven approaches identify ADHD profiles with distinct cognitive profiles based on the dual-deficit patterns revealed by the WCST? Do these cognition-based subtypes differ systematically in clinical manifestations (SNAP-IV and ADHD clinical subtypes)?

Based on existing theories and previous empirical evidence, we proposed three hypotheses. First, children with ADHD will show coexisting yet relatively independent dual executive function deficits on the WCST, with a moderate correlation (r < 0.5) between inhibitory control and cognitive flexibility deficits, supporting their relative independence (Hypothesis 1). Second, inhibitory control deficits will show significant improvement with age, whereas cognitive flexibility deficits will show less pronounced age-related change, reflecting potentially different developmental sensitivities of these two components (Hypothesis 2). Third, based on the severity of these deficits, at least three ADHD subtypes with distinct cognitive phenotypes can be reliably identified—dual deficit, inhibition dominant, and mild deficit—with the dual deficit subtype expected to be associated with more extensive clinical symptoms and more severe daily functional impairment (Hypothesis 3).

The present study makes three primary contributions. First, by using trial-level GLMM analysis, it provides a direct test of whether inhibitory control and cognitive flexibility deficits are relatively independent—a question central to the theoretical debate between single core deficit and multi-component models. Second, by examining age-related changes within the 7–12 age range, it clarifies whether these deficits show detectable improvement during this critical developmental period, informing prognosis and intervention timing. Third, based on the severity of these deficits, distinct ADHD subtypes with different cognitive phenotypes can be reliably identified—including a dual deficit subtype and a mild deficit subtype—with the dual deficit subtype expected to be associated with more extensive cognitive impairment.

## 2 Materials and method

### 2.1 Participants

The final analytic sample consisted of 281 children with ADHD (198 males, 70.5%; 83 females, 29.5%). Age ranged from 7 to 12 years (M = 9.8 years, SD = 1.7 years), with the following age distribution based on the analytic sample: 7 years (n = 37, 13.2%), 8 years (n = 42, 14.9%), 9 years (n = 41, 14.6%), 10 years (n = 43, 15.3%), 11 years (n = 52, 18.5%), and 12 years (n = 66, 23.5%). The original sample comprised 305 children, of whom 24 (7.9%) were excluded due to data quality issues (task completion rate below 80% or random response patterns) and classified as the NA subtype. Detailed exclusion criteria are provided in the Data Preprocessing section.

All participants were recruited from the child psychiatry outpatient clinic of a tertiary hospital between 2023 and 2024.

Inclusion criteria were: (1) age 7–12 years, any gender; (2) meeting DSM-5 diagnostic criteria for ADHD, independently confirmed by two child psychiatrists; (3) ability to complete the computerized WCST and related assessment tasks; and (4) written informed consent obtained from both caregivers and participants.

Exclusion criteria included: (1) comorbid severe neurological disorders (e.g., epilepsy, organic brain disease), psychiatric disorders (e.g., autism spectrum disorder, schizophrenia), or other medical conditions (e.g., thyroid dysfunction); (2) intellectual disability (Raven’s Standard Progressive Matrices score below 70); (3) use of medications affecting cognitive function (e.g., antipsychotics, antiepileptics) within the past three months; and (4) poor task engagement during data collection (task completion rate below 80%) or missing data exceeding 10%.

Clinical characteristics. ADHD symptom severity was assessed using the SNAP-IV rating scale (Swanson, Nolan, and Pelham Rating Scale-IV), completed by parents. The scale consists of two subscales: inattention (9 items) and hyperactivity/impulsivity (9 items), each rated on a 0–3 scale. In the present sample, the mean score for the inattention subscale was 14.81 (SD = 5.39), and the mean score for the hyperactivity/impulsivity subscale was 10.55 (SD = 6.07), with a mean total item score of 1.41 (SD = 0.58). Nonverbal reasoning ability was assessed using Raven’s Standard Progressive Matrices (SPM). The mean score of the sample was 96.19 (SD = 23.78), indicating that the overall intellectual functioning of the participants was within the normal range. All participants were first-visit, treatment-naïve children who had never received ADHD-related medications; thus, potential medication effects on cognitive performance could be ruled out (see **Supplementary Table 1**).

Regarding ADHD clinical presentations based on DSM-5 criteria, the sample included predominantly inattentive, predominantly hyperactive-impulsive, and combined presentations. The proportions were consistent with typical epidemiological patterns of childhood ADHD.

Sample size calculation. Based on pilot study results (effect size d = 0.35), a minimum required sample size of 252 was calculated using G*Power 3.1 (α = 0.05, power = 0.85). The final analytic sample of 281 exceeded this requirement, ensuring adequate statistical power.

Note on analytic sample. All primary analyses—including GLMM, linear regressions, and Gaussian mixture modeling—were conducted on the quality-controlled analytic sample of 281 participants. A sensitivity analysis comparing included and excluded participants on demographic characteristics and WCST outcomes is provided in **Supplementary Table 3**.

### 2.2 WCST and procedure

#### 2.2.1 Instrument

A computerized version of the Wisconsin Card Sorting Test (WCST) was used to assess executive function, following the standardized manual by Heaton et al. (11). The test interface displayed four stimulus cards fixed at the top of the screen. From left to right, the stimulus cards were: one red triangle, two green stars, three yellow crosses, and four blue circles. Response cards were presented sequentially at the bottom of the screen and consisted of various combinations of color (red, green, yellow, blue), shape (triangle, star, cross, circle), and quantity (1–4). The test employed two standard decks of 64 response cards each, totaling 128 trials. A schematic diagram of the computerized task interface is provided in **Supplementary Figure 1** (Supplementary Material).

#### 2.2.2 Task structure

The WCST consisted of four sequential task phases, each defined by a hidden sorting rule. The full test included a total of 128 trials with no additional breaks:

Baseline (color rule): The initial sorting rule was color. Participants had to match each response card to the stimulus card sharing the same color. This phase served as the baseline for assessing color-rule performance.

Shape rule: After 10 consecutive correct responses under the color rule, the sorting rule automatically shifted to shape without explicit notification. Participants discovered through feedback that they now needed to match by shape rather than color.

Number rule: After 10 consecutive correct responses under the shape rule, the rule shifted to number. Participants then had to match by quantity.

Color return: After 10 consecutive correct responses under the number rule, the rule shifted back to color. This return-to-color phase enabled assessment of whether performance recovered to baseline levels.

Feedback and transition logic. After each match, the computer provided immediate feedback (“correct” or “incorrect”). Participants used this feedback to infer the current sorting rule. The rule changed automatically after every 10 consecutive correct responses without any explicit notification. No time limit was imposed for individual responses, but participants could not proceed to the next trial until a response was made. An experimenter remained present throughout the session to ensure task comprehension but provided no hints regarding the sorting rules.

**Scoring logic.** All trials were coded dichotomously as correct or incorrect for trial-level analysis. Core indices including perseverative error rate (PER) and shape rule disadvantage (SRD) were calculated offline following the scoring criteria proposed by Heaton et al. (11).

### 2.3 Core indices

Based on Heaton et al. (11) scoring criteria, the following core indices were calculated:

Perseverative error rate (PER): The proportion of errors in which the participant continued to apply a previously correct rule after a rule switch. An error was classified as perseverative if the response would have been correct under the immediately preceding rule. This index reflects inhibitory control—the ability to suppress outdated response tendencies (3, 4).

Shape rule disadvantage (SRD): The difference between accuracy under the shape rule and accuracy under the baseline color rule, calculated as SRD = Accuracy_Shape – Accuracy_Color. This subtraction-based approach provides a within-subject control for individual baseline differences in color-rule performance, thereby isolating the cognitive cost of switching from the initially learned color dimension to the shape dimension. A positive SRD value indicates poorer performance on shape rules, reflecting increased difficulty in rule switching.

Conceptually, SRD captures set-shifting ability, which serves as a core component of cognitive flexibility in accordance with the unity and diversity model of executive functions proposed by Miyake et al. (6). The logic of isolating switch costs through subtraction of a baseline condition is methodologically analogous to the approach used in task-switching paradigms, where switch costs are calculated as the performance difference between task-switch and task-repeat trials (7). In the WCST context, the transition from color to shape constitutes the first and most critical set-shift, as participants must disengage from an already-established sorting dimension. Because color is perceptually more salient than shape, shifting from color to shape imposes greater cognitive demands and is therefore particularly sensitive to individual differences in set-shifting ability.

However, it should be noted that WCST performance involves multiple overlapping cognitive processes, including working memory, abstraction, feedback processing, and inductive reasoning (13). SRD should therefore be interpreted as a behavioral indicator of cognitive flexibility within the WCST context, rather than a process-pure measure of set-shifting ability. Despite this limitation, SRD offers an advantage over traditional aggregate indices such as total errors or categories completed by providing a more specific and computationally transparent measure of rule-switching difficulty that controls for individual baseline performance.

Overall accuracy: The proportion of correct responses across all 128 trials.

### 2.4 Data preprocessing and statistical analysis

All statistical analyses were conducted using R software (version 4.3.0). Mixed-effects modeling was performed using the `lme4` package, and Gaussian mixture model clustering was conducted using the `mclust` package. Raw WCST data were first transformed from wide format to long format to facilitate trial-level modeling and analysis. Participants were excluded based on the criteria described in Section 2.1. The final analytic sample comprised 281 children with ADHD.

Generalized linear mixed model (GLMM). To identify the core factors influencing WCST performance, a GLMM was constructed. The dependent variable was trial-level response correctness (binary, coded as 1 = correct, 0 = incorrect). Fixed effects included age (centered around the sample mean), gender (male as reference), and task phase (Baseline/color baseline as reference; ShapeRule/shape rule; NumberRule/number rule; ColorReturn/return to color). Participant ID was included as a random intercept to account for within-subject correlations across trials. The model was fitted using the `glmer` function with the bobyqa optimizer. Model goodness-of-fit was assessed using marginal and conditional R² following Nakagawa and Schielzeth (19). Variance inflation factors (VIF) were examined to confirm the absence of multicollinearity among predictors (all VIFs < 2.0).

Age-related differences analysis. Linear regression analyses were conducted separately for PER and SRD as a function of age. An age × deficit type interaction term was included in a combined model (with deficit type coded as a binary variable) to test whether the two deficits showed different age-related change patterns. Effect sizes were reported as Cohen’s *f*².

Cognitive profile identification. Gaussian mixture model (GMM) clustering was employed to identify cognitive profiles based on PER and SRD. Model comparison was conducted for two-, three-, and four-class solutions using both Bayesian Information Criterion (BIC) and Akaike Information Criterion (AIC). The optimal number of classes was determined primarily by the lowest BIC value. Between-profile differences in PER, SRD, and overall accuracy were examined using one-way ANOVA with Tukey HSD *post-hoc* tests. Effect sizes were reported as η².

### 2.5 Ethics statement

This study was approved by the Ethics Committee of Wenzhou Seventh People’s Hospital (Approval No: EC-KY-202408). Written informed consent was obtained from all participants and their legal guardians prior to data collection. All procedures were conducted in accordance with the Declaration of Helsinki.

## 3 Result

### 3.1 Data preprocessing and quality control

After quality-control exclusion (see Section 2.4), the final analytic sample comprised 281 children with ADHD, representing 92.1% of the original 305 participants.

### 3.2 Overall WCST performance

**Figure 1:**
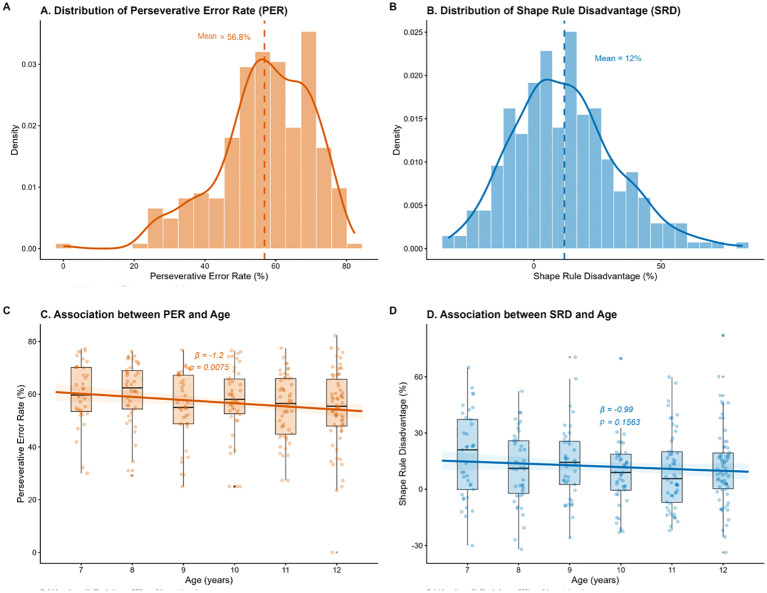
Core cognitive indicators: distribution and age associations. **(A)** Distribution of perseverative error rate (PER). Values near zero reflect participants with very few color-rule trials before the first rule switch. **(B)** Distribution of shape rule disadvantage (SRD). **(C)** Association between PER and age. Solid line represents the linear fit; shaded area indicates the 95% confidence interval. **(D)** Association between SRD and age. Boxplots display the distribution at each age group. All panels are based on the quality-controlled analytic sample of *N* = 281.

Across a total of 37,215 trials, children with ADHD achieved an overall accuracy rate of 60.3% (SD = 14.2%). The perseverative error rate, an index of inhibitory control, was 56.8% (SD = 13.2%), and the shape rule disadvantage, an index of cognitive flexibility, was 12.0% (SD = 20.3%). The two core cognitive indices showed a weak positive correlation (*r* = 0.245, *p* < 0.001), with a shared variance of only 6.0%. This low correlation suggests that PER and SRD capture partially independent aspects of executive dysfunction, consistent with the view that inhibitory control and cognitive flexibility are separable but not entirely unrelated components (6). Detailed performance metrics by age group are presented in **Table 1** and **Figure 1**.

**Table 1:** Core WCST performance metrics by age group.

Age group	n	Perseverative error rate (%)	Shape rule disadvantage (%)	Overall accuracy (%)
7 years	37	60.4 ± 11.7	17.8 ± 22.9	55.4 ± 13.8
8 years	42	60.3 ± 12.0	11.3 ± 19.5	53.7 ± 14.1
9 years	41	55.2 ± 12.9	14.1 ± 19.7	62.3 ± 12.7
10 years	43	57.4 ± 13.3	9.2 ± 18.1	60.6 ± 12.7
11 years	52	55.1 ± 12.2	9.5 ± 20.6	63.3 ± 14.0
12 years	66	54.5 ± 14.8	11.7 ± 20.6	63.7 ± 14.8
Total	281	56.8 ± 13.2	12.0 ± 20.3	60.3 ± 14.2

All values are based on the quality-controlled analytic sample of 281 participants. PER = perseverative error rate; SRD = shape rule disadvantage.

### 3.3 Generalized linear mixed model analysis

To identify the core factors influencing WCST performance, a generalized linear mixed model (GLMM) was constructed. The dependent variable was trial-level response correctness (binary). Fixed effects included age (centered), gender, and task phase (TaskPhase: Baseline/color baseline, ShapeRule/shape rule, NumberRule/number rule, ColorReturn/return to color). Participant ID was included as a random intercept.

**Figure 2:**
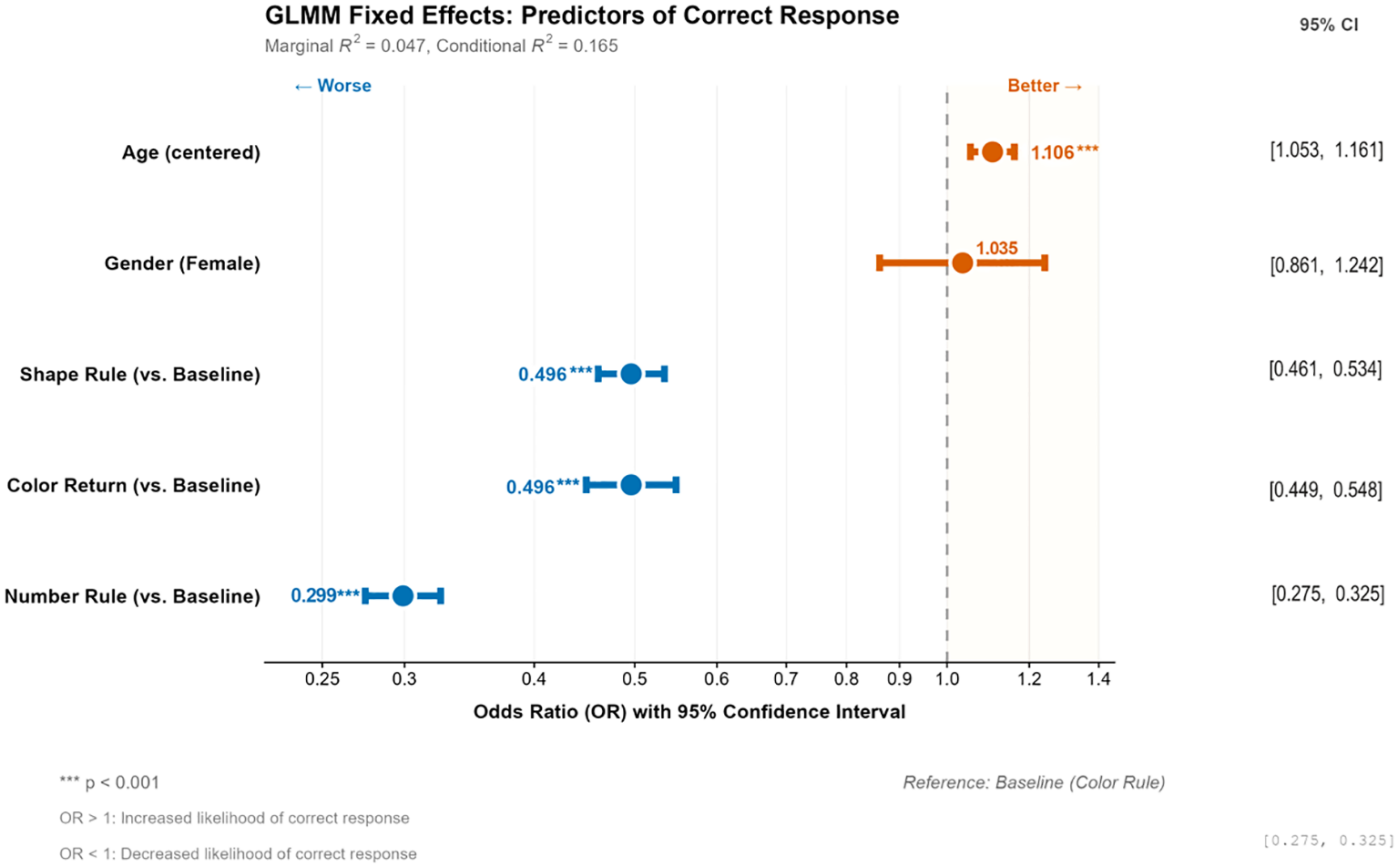
GLMM = generalized linear mixed model. The model was fitted with a binomial distribution and logit link function using the glmer function in the lme4 package (R version 4.3.0). Fixed effects: age (centered around the sample mean),gender (male = 0,female = 1),and task phase (Baseline as reference). Random effect: participant ID (random intercept). OR = odds ratio; CI = confidence interval. Model diagnostics: marginal R² = 0.047,conditional R² = 0.165; all variance inflation factors (VIF) < 2.0,indicating no multicollinearity. N = 281 participants,37,215 trials.

The model yielded a marginal R² of 0.047 and a conditional R² of 0.165, indicating that fixed effects explained approximately 4.7% of the variance in response accuracy, with an additional 11.8% explained by individual differences, for a total of 16.5% variance accounted for (see **Table 2**, **Figure 2**).

**Table 2:** GLMM fixed effect results.

Predictor	*β*	SE	*z*	*p*	OR	95% CI
Intercept	1.184	0.059	19.91	<0.001	3.268	[2.909, 3.673]
Age (centered)	0.100	0.025	4.03	<0.001	1.106	[1.053, 1.161]
Gender (female)	0.034	0.093	0.36	0.716	1.035	[0.861, 1.242]
Shape rule (vs. baseline)	-0.701	0.038	-18.53	<0.001	0.496	[0.461, 0.534]
Number Rule (vs. Baseline)	-1.207	0.043	-28.32	<0.001	0.299	[0.275, 0.325]
Color Return (vs. Baseline)	-0.701	0.051	-13.79	<0.001	0.496	[0.449, 0.548]

GLMM = generalized linear mixed model. The model was fitted with a binomial distribution and logit link function using the glmer function in the lme4 package (R version 4.3.0). Fixed effects included age (centered around the sample mean),gender (male as reference),and task phase (Baseline as reference). A random intercept was included for participant ID. OR = odds ratio; CI = confidence interval. Model diagnostics: marginal R^2^ = 0.047,conditional R^2^ = 0.165; all variance inflation factors (VIF) < 2.0,indicating no multicollinearity. N = 281 participants,37,215 trials.

For each one-year increase in age, the odds of a correct response increased by 10.6% (OR = 1.106, *p* < 0.001).

Under the shape rule, the probability of a correct response was 50.4% lower than under the baseline color rule (OR = 0.496, *p* < 0.001).

Under the number rule, the probability of a correct response was 70.1% lower than under the baseline color rule (OR = 0.299, *p* < 0.001).

Upon returning to the color rule, performance remained 50.4% lower than baseline (OR = 0.496, *p* < 0.001), suggesting the presence of fatigue or sustained attention deficits in later task stages.

Gender effects were not statistically significant (*p* = 0.716).

### 3.4 Age-related differences analysis

To examine age-related changes in inhibitory control and cognitive flexibility deficits, linear regression analyses were conducted separately for perseverative error rate (PER) and shape rule disadvantage (SRD) as a function of age.

Inhibitory control deficit (PER): PER showed a significant improvement trend with increasing age (β = -1.204, SE = 0.447, *t* = -2.70, *p* = 0.008, 95% CI [-2.083, -0.325]), corresponding to an average decrease of 1.2 percentage points in perseverative error rate per year of age. The effect size was Cohen’s *f*² = 0.026, indicating a weak effect.

Cognitive flexibility deficit (SRD): The improvement trend with age for SRD was not statistically significant (β = -0.988, SE = 0.695, *t* = -1.42, *p* = 0.156, 95% CI [-2.357, 0.380]). The effect size was Cohen’s *f*² = 0.007, which is negligible.

**Figure 3:**
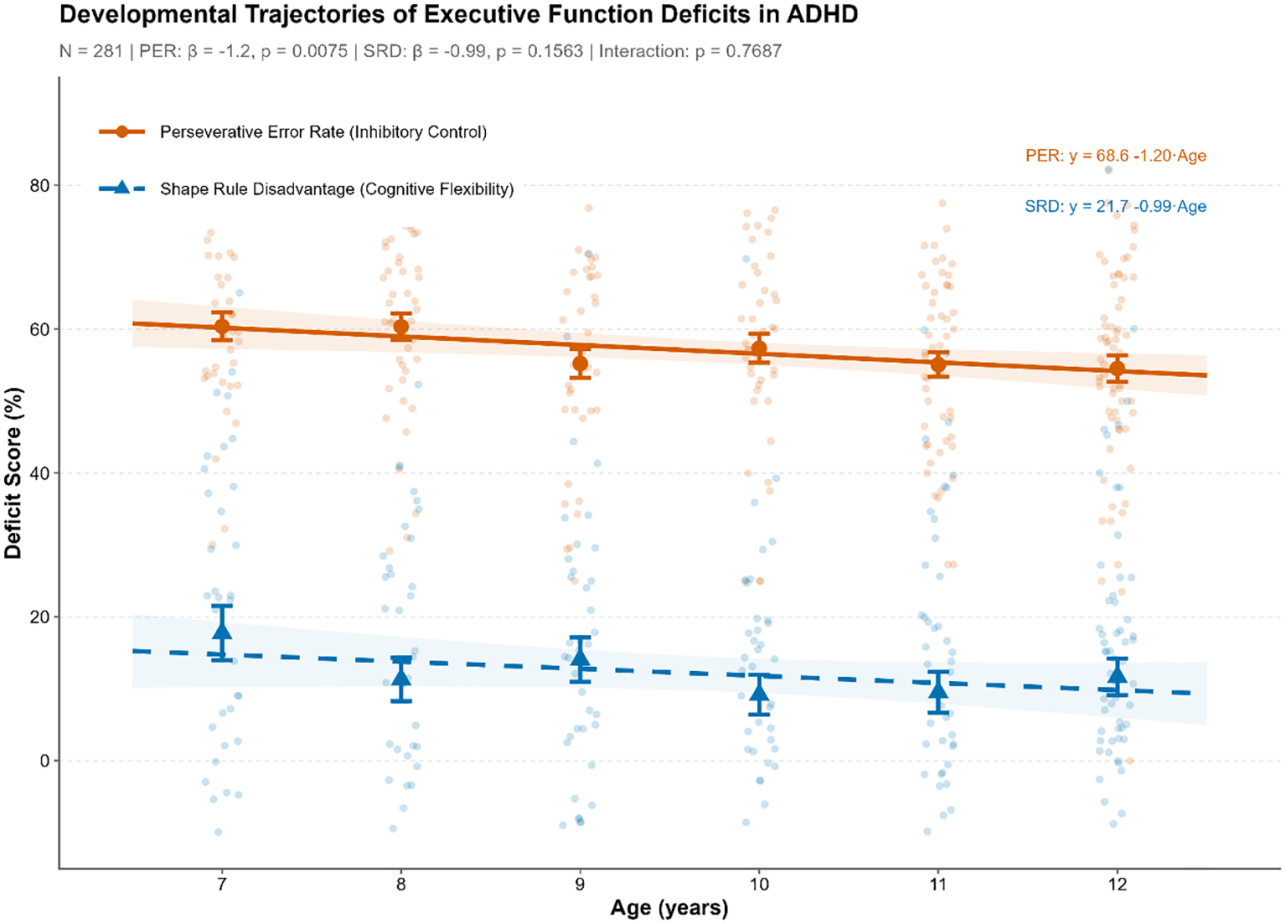
Age-related change patterns of inhibitory control and cognitive flexibility deficits. Solid lines represent linear fits; shaded areas indicate 95% confidence intervals. Error bars represent mean ± SEM. PER = perseverative error rate; SRD = shape rule disadvantage. N = 281.

Interaction test: The age × deficit type interaction was not statistically significant (β = 0.216, SE = 0.826, *p* = 0.794), indicating that the developmental slopes of the two deficits did not differ significantly (see **Figure 3**).

### 3.5 Identification of cognitive profiles

To identify cognitive profiles of ADHD based on inhibitory control (PER) and cognitive flexibility (SRD), Gaussian mixture model (GMM) clustering was employed. Model comparison was conducted for two-, three-, and four-class solutions using both Bayesian Information Criterion (BIC) and Akaike Information Criterion (AIC). The two-class solution yielded the lowest BIC (-4713.8), supporting its selection as the optimal model. Although AIC continued to decrease with additional classes (two-class: 4684.7; three-class: 4657.1; four-class: 4647.4), BIC—which imposes a stronger penalty on model complexity—clearly favored the two-class solution. The three-class solution produced a small intermediate subgroup without clear theoretical interpretation, and the four-class solution showed a higher BIC than the two-class solution, suggesting overfitting. Complete model comparison statistics are provided in **Supplementary Table 2**.

The two-class solution identified two preliminary cognitive profiles with distinct impairment patterns:

Mild deficit subtype (*n* = 107, 38.1%): This subtype was characterized by a relatively low perseverative error rate (47.9% ± 15.3%), a shape rule disadvantage near zero (1.2% ± 11.4%), and a relatively high overall accuracy (68.5% ± 14.3%), representing a phenotype with relatively preserved cognitive function.

**Figure 4:**
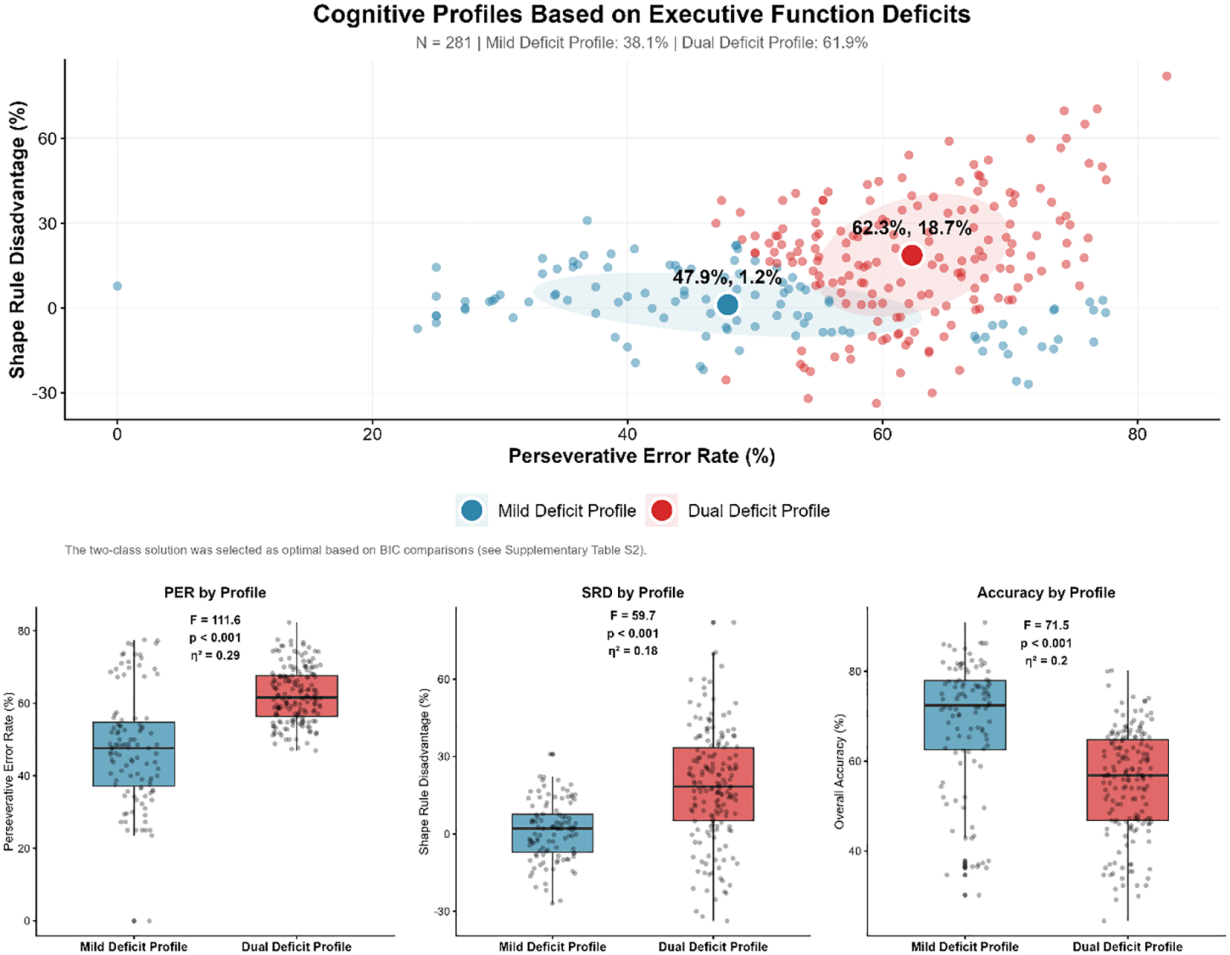
Cognitive profiles identified by Gaussian mixture modeling. **(A)** Scatter plot of PER and SRD with 95% confidence ellipses for each profile. **(B)** Boxplots comparing PER,SRD,and overall accuracy between profiles. The two-class solution was selected as optimal based on BIC and AIC comparisons against three- and four-class alternatives (see **Supplementary Table 2** for model comparison statistics).

Dual deficit subtype (*n* = 174, 61.9%): This subtype was characterized by a higher perseverative error rate (62.3% ± 7.6%), a pronounced shape rule disadvantage (18.7% ± 21.7%), and lower overall accuracy (55.3% ± 11.6%), representing a phenotype with concurrent impairments in both inhibitory control and cognitive flexibility (see **Table 3**, **Figure 4**).

**Table 3:** Core characteristics of cognitive profiles.

Indicator	Mild deficit (n=107)	Dual deficit (n=174)	*F*	*p*	*η²*
Perseverative error rate (%)	47.9 ± 15.3	62.3 ± 7.6	111.6	<0.001	0.29
Shape rule disadvantage (%)	1.2 ± 11.4	18.7 ± 21.7	59.7	<0.001	0.18
Overall accuracy (%)	68.5 ± 14.3	55.3 ± 11.6	71.5	<0.001	0.20

*Post-hoc* tests (Tukey HSD) confirmed that the differences between the two cognitive profiles were statistically significant across all indicators (all p < 0.001). The effect sizes (η²) for profile differentiation ranged from 0.18 to 0.29, representing moderate to strong clinical effects.

## 4 Discussion

This study employed trial-level fine-grained analysis of Wisconsin Card Sorting Test (WCST) performance in children with ADHD to systematically investigate the relative independence of dual executive function deficits, age-related change patterns, and clinical profile characteristics. The main findings were as follows: children with ADHD exhibited relatively independent dual deficits in inhibitory control and cognitive flexibility on the WCST; between the ages of 7 and 12 years, inhibitory control showed a significant negative cross-sectional association with age, whereas changes in cognitive flexibility could not be reliably identified statistically; and based on the severity of these dual deficits, two ADHD cognitive profiles with distinct cognitive phenotypes were identified, with the dual deficit profile associated with more extensive cognitive impairment. Each of these findings is discussed in turn below.

### 4.1 Relative independence of dual deficits

The first core finding of this study is that children with ADHD exhibit relatively independent dual deficits in inhibitory control and cognitive flexibility on the WCST. Specifically, children with ADHD not only showed a high rate of perseverative errors—a classic index of inhibitory control deficits (3, 4)—but, more critically, exhibited specific processing difficulties under the shape rule. Generalized linear mixed model analysis revealed that, after controlling for covariates including age, gender, and task phase, the shape rule still exerted a significant main effect on response accuracy, indicating that cognitive flexibility deficits are not merely a secondary derivative of inhibitory control deficits but exert effects independent of demographic variables and task progression. Furthermore, perseverative error rate and shape rule disadvantage showed only a weak positive correlation, with very limited shared variance, which further aligns with predictions of multi-component executive function frameworks at the individual level.

This finding converges with evidence from other cognitive paradigms. Using a task-switching paradigm, Cepeda and colleagues identified specific cognitive flexibility impairments in children with ADHD, characterized by significantly increased switch costs that were correlated with, yet relatively independent from, inhibitory control deficits (7). Unlike the WCST, the task-switching paradigm employs explicitly pre-specified rules without requiring exploration, thereby providing a purer measure of set-shifting ability. The consistent findings across these two paradigms offer cross-paradigm validation for the independence of cognitive flexibility deficits. Moreover, large-scale meta-analyses have reported that executive dysfunction in ADHD is not a unidimensional impairment but rather involves composite deficits across multiple domains, including inhibitory control, cognitive flexibility, and working memory (4).

These findings are broadly consistent with multi-component frameworks of executive function, such as the unity/diversity model proposed by Miyake and colleagues. Using latent variable analysis, this model first established that inhibitory control, cognitive flexibility, and working memory updating are three relatively independent yet interrelated cognitive components, each with a distinct cognitive structure (6). It should be noted that SRD, like all WCST-derived indices, reflects the combined influence of multiple cognitive processes rather than providing a process-pure measure of set-shifting. Nevertheless, this limitation does not undermine the pattern of relative independence between the two deficits. The low correlation observed in the present study conforms to the model’s prediction that independent components should exhibit correlations below moderate levels. In contrast, Barkley’s model conceptualizes cognitive flexibility impairments as a secondary consequence of a core inhibitory control deficit (3), predicting that the two should be highly correlated and change synchronously. The data pattern observed in the present study does not align with the latter prediction, suggesting that the data are more compatible with the framework of partially independent cognitive components.

The unity/diversity model of executive function has received robust support from neuroimaging research. A review by Rubia and colleagues indicated that inhibitory control relies primarily on fronto-striatal circuitry, particularly the right inferior frontal cortex and supplementary motor area, whereas cognitive flexibility is closely associated with coordinated activation of the anterior cingulate-parietal network and dorsolateral prefrontal cortex. Meta-analyses of functional magnetic resonance imaging studies have further confirmed that these two neural circuits exhibit relatively independent patterns of aberrant activation in individuals with ADHD (5). This correspondence is broadly consistent with Badre and Wagner’s theory of prefrontal functional specialization, which posits that the dorsolateral prefrontal cortex is primarily responsible for conflict resolution, maintenance of rule representations, and adaptive updating, whereas the ventrolateral prefrontal cortex is more involved in the selective inhibition of stimulus-response associations (20). Although prior neuroimaging work identifies dissociable frontostriatal and frontoparietal networks for inhibitory control and set-shifting, it should be noted that the present study did not collect neuroimaging data, and these neural interpretations remain speculative. The present behavioral findings align broadly with multi-component executive function perspectives in ADHD, without constituting a formal test of the full multiple deficit framework. However, the limitations of WCST-derived indices as behavioral indicators should be acknowledged, as discussed in Section 2.3.

### 4.2 Patterns of age-related change

The second core finding concerns the cross-sectional association between age and the two executive function deficits. Within the 7–12 year age range, PER showed a significant negative association with age, whereas SRD did not. However, the age × deficit type interaction was not significant (*p* = 0.794). This means the data do not support the conclusion that the two deficits follow different developmental patterns. The most prudent interpretation is that PER was negatively associated with age in this sample, while the association between SRD and age could not be statistically distinguished from zero.

The effect sizes were small (PER: *f*² = 0.026; SRD: *f*² = 0.007), and the cross-sectional design precludes inferences about within-individual developmental change. These age-related associations represent between-subject differences that may reflect cohort effects rather than maturational processes.

Despite the non-significant interaction, this asymmetric result—PER showing a significant age association and SRD showing a negligible one—conceptually resonates with evidence from other paradigms. Qian and colleagues found that inhibitory control deficits in ADHD no longer differed significantly from controls by early adolescence, whereas cognitive flexibility deficits persisted into mid-adolescence (21). Task-switching studies have also reported that cognitive flexibility impairments respond differently to pharmacological intervention compared with inhibitory control deficits (7). These correlational cross-sectional findings raise the speculative hypothesis that executive subcomponents may mature at different rates, a claim requiring longitudinal validation (22).

Should this hypothesis prove valid, it would be broadly consistent with multi-component frameworks of executive function, such as the unity/diversity model (6). According to this model, inhibitory control and cognitive flexibility, as relatively independent components, each possess distinct cognitive structures and neural substrates. If they are indeed independent, they could be governed by different neurodevelopmental processes, though this remains provisional. Developmental cognitive neuroscience provides a plausible but speculative framework for this inference: fronto-striatal circuitry associated with inhibitory control undergoes continued structural remodeling throughout childhood and adolescence (23), whereas functional integration of the frontoparietal network underlying cognitive flexibility may require a longer timescale before detectable behavioral improvements emerge (24). However, the present study did not collect neuroimaging data, and these neural interpretations remain speculative.

It is important to emphasize that the above interpretation remains speculative in the context of the present study. Future research employing longitudinal designs should directly test the developmental timing of different executive function components.

### 4.3 Identification of cognitive profiles and clinical implications

The third core finding of this study is that, based on the severity of dual deficits in inhibitory control and cognitive flexibility, two ADHD cognitive profiles with distinct cognitive phenotypes can be identified. This finding addresses a long-standing question in ADHD research: does cognitive heterogeneity reflect continuous variation in a single core deficit, or qualitative differences in how multiple cognitive impairments combine? Using a data-driven Gaussian mixture model clustering approach, this study identified two preliminary cognitive profiles. The mild deficit profile is characterized by relatively mild impairments in inhibitory control and largely intact cognitive flexibility, whereas the dual deficit profile is characterized by concurrent impairments in both inhibitory control and cognitive flexibility, accounting for over sixty percent of the sample and representing the predominant cognitive phenotype in the ADHD population. The two profiles differed significantly across all core cognitive indices, indicating that this classification possesses substantive clinical discriminative validity.

The identification of this profile structure provides critical evidence for evaluating competing theoretical models. If executive dysfunction in ADHD were attributable to a single core inhibitory control deficit, as proposed by Barkley’s model (3), then children with ADHD should predominantly manifest continuous variation in the severity of inhibitory control impairment, rather than a qualitative differentiation pattern in which “some children show impaired inhibitory control but intact cognitive flexibility, while others show impairments in both.” Yet, the profile pattern observed here aligns broadly with predictions from multi-component executive function frameworks, such as the unity/diversity model (6)—according to which inhibitory control and cognitive flexibility, as relatively independent cognitive components, can be impaired singly or in combination. In other words, cognitive heterogeneity in ADHD is manifested not only in “quantitative” differences in impairment severity, but also in “qualitative” differences in impairment patterns (25).

From a clinical perspective, this profile classification suggests that comprehensive assessment may benefit from capturing multiple executive function dimensions (26). Children with the mild deficit profile experience primary difficulties in the inhibitory control domain and may respond better to interventions targeting inhibitory control (e.g., response inhibition training); whereas children with the dual deficit profile, who exhibit concurrent impairments in cognitive flexibility, may require comprehensive intervention strategies that address both rule switching and cognitive set updating. Moreover, the fact that the dual deficit profile accounts for over sixty percent of the sample underscores the importance of attending to cognitive flexibility in clinical assessment—traditional neuropsychological assessments of ADHD have often centered on inhibitory control, potentially underestimating the prevalence and clinical significance of cognitive flexibility deficits (17). Importantly, the identified clusters should be interpreted with caution. Because the current analysis is based on a single cross-sectional sample without an independent validation cohort, the identified cognitive profiles represent preliminary, data-driven groupings rather than definitively established clinical profiles. The observed clusters may reflect dimensional differences in executive dysfunction severity, rather than qualitatively distinct categorical profiles (25). Future research employing longitudinal designs, independent validation samples, and clinical outcome data will be essential to determine the stability, generalizability, and clinical utility of these preliminary cognitive profiles.

Prior neuroimaging work documents heterogeneous brain connectivity in ADHD (27), and recent transdiagnostic connectome evidence suggests that distinct structural neural subtypes may underlie similar behavioral profiles of inattention and hyperactivity in children with clinically elevated symptoms (28). Nevertheless, the current WCST-derived cognitive profiles cannot be linked to specific neural substrates in the absence of imaging data; neural correlates of these two profiles await future multimodal research. The present analysis only captures variation within cool executive function indices measured via WCST, and cannot inform cool/hot dual-pathway mechanisms; simultaneous assessment of reward- and emotion-related hot executive function is required to test links to this broader framework (9).

Beyond the executive-function heterogeneity observed in the present WCST analysis, contemporary theoretical frameworks frame ADHD as a multidimensional neurodevelopmental condition modulated by interacting biological systems, among which circadian and sleep-wake regulatory processes stand out (29, 30). Circadian clock dysregulation has been linked to disrupted sustained attention and impulse regulation, producing heterogeneous clinical presentations that may be partially independent of the cool executive deficits captured here. While the present cognitive profile classification is limited to WCST performance, integrating sleep and circadian phenotypes alongside executive profiles may yield more holistic models of ADHD individual differences for future investigation.

It is worth noting that the revelation of these three core findings was substantially enabled by the methodological strategy employed in this study. As noted in the Introduction, existing WCST research has been constrained by a lack of integrative analytical perspectives, with most studies treating multiple traditional indices as isolated outcome measures, lacking systematic examination of rule-specific effects and dynamic patterns across task phases (12, 13). By employing GLMM to fully utilize all trial-level data and simultaneously examine the interactive effects of age, rule type, and task phase, this study’s fine-grained trial-level analysis brought into relief rule-specific effects and cognitive profile differentiation that traditional methods would have failed to capture. This methodological choice suggests that future executive function research may benefit from attending more closely to the “profile” rather than merely the “total score” of cognitive processes—focusing on “when and where” difficulties arise, rather than simply “whether” they exist.

## 5 Conclusions

In summary, this study employed fine-grained trial-level analysis of WCST performance in children with ADHD to yield three core findings.

First, children with ADHD exhibit relatively independent dual deficits in inhibitory control and cognitive flexibility, broadly consistent with multi-component frameworks of executive function in the ADHD population.

Second, within the specific developmental window of 7 to 12 years of age, inhibitory control shows a significant negative cross-sectional association with age, whereas the cross-sectional association between cognitive flexibility and age could not be reliably identified statistically.

Third, based on the severity of these dual deficits, two preliminary cognitive profiles were identified, warranting further validation through longitudinal and independent cohort studies, with the dual deficit profile accounting for over sixty percent of the sample and associated with more extensive cognitive impairment.

These findings deepen our understanding of cognitive heterogeneity in ADHD, suggesting that inhibitory control and cognitive flexibility, as relatively independent cognitive components, exhibit distinct patterns of impairment combination in this population. The results suggest that comprehensive assessment of ADHD should consider both inhibitory control and cognitive flexibility, as focusing exclusively on one domain may provide an incomplete picture of a child’s executive function profile. Building on these findings, future research should employ longitudinal designs to examine the stability and predictive utility of these cognitive profiles over time, and examine how these profiles relate to neural mechanisms, clinical outcomes, and intervention responses.

### 5.1 Theoretical contributions

This study offers within-task behavioral patterns that align broadly with multi-component executive frameworks rather than single core deficit perspectives, and the two distinct cognitive profiles identified further illustrate how such multi-component frameworks can explain individual clinical differences.

### 5.2 Practical implications

The present findings suggest that comprehensive ADHD assessment may benefit from evaluating both inhibitory control and cognitive flexibility, as focusing on a single domain may provide an incomplete picture of executive function in a substantial proportion of children. Future interventional work may explore whether tailored training focusing on distinct executive weaknesses could benefit different cognitive profiles; no clinical intervention conclusions can be drawn from the current cross-sectional WCST data.

### 5.3 Limitations and future directions

Despite the important findings of this study, several limitations should be acknowledged and addressed in future research.

First, the cross-sectional design constrains causal inferences regarding age-related differences. The age-related associations reported in this study are based on comparisons across children of different ages and may be confounded by cohort effects. Longitudinal studies that track the same group of children with ADHD over time are needed to verify whether the findings reported here reflect genuine intra-individual developmental change.

Second, the present study did not include a healthy control group or a non-ADHD clinical control group. Consequently, the observed cognitive profiles cannot be interpreted as ADHD-specific; they may also be present in other neurodevelopmental or psychiatric conditions. The current design can describe heterogeneity within children with ADHD, but cannot determine whether the identified patterns distinguish ADHD from typical development or from other clinical populations. Future studies should include both healthy and clinical control groups to assess the specificity of these cognitive profiles.

Third, the absence of neuroimaging data limits the ability to directly link cognitive profiles with neural circuitry. Establishing direct associations between cognitive profiles and neural circuits is a critical step toward validating the biological basis of the classification system. Future research should integrate multimodal neuroimaging techniques to explore the neurobiological substrates underlying the different cognitive profiles.

Fourth, the representativeness of the sample is limited. The sample was primarily drawn from a single-center clinical outpatient setting, which may restrict the generalizability of the findings. Future studies should incorporate community-recruited samples and systematically collect environmental variables such as socioeconomic status and family educational resources to evaluate the moderating role of environmental factors on cognitive development.

Fifth, a subset of participants was excluded due to data quality concerns. These excluded participants showed markedly lower overall accuracy and higher perseverative error rates compared with the analytic sample (see **Supplementary Table 3**), suggesting they represent a subgroup with extremely poor task performance or difficulty comprehending task instructions. Their exclusion may limit the generalizability of findings to the most severely affected children with ADHD. Future research should refine task instructions and procedures to minimize data loss and better characterize the cognitive profiles of these children.

## Data Availability

The raw data supporting the conclusions of this article will be made available by the authors, without undue reservation.

## Supplementary material

Supplementary Figure 1 Schematic diagram of the computerized Wisconsin card sorting test (WCST) interface.

Supplementary Table 1 Demographic and clinical characteristics of the ADHD sample (N = 281). SNAP-IV = Swanson, Nolan, and Pelham Rating Scale-IV; SPM = Standard Progressive Matrices. All participants were first-visit, treatment-naïve children with ADHD

Supplementary Table 2 Gaussian mixture model comparison for two-, three-, and four-class solutions. BIC = Bayesian Information Criterion; AIC = Akaike Information Criterion. Lower BIC values indicate better model fit. The two-class solution was selected as optimal because it yielded the lowest BIC. The three-class solution produced a small intermediate subgroup (n = 48) without clear theoretical interpretation, and the four-class solution showed a higher BIC than the two-class solution, suggesting overfitting.*

Supplementary Table 3 Sensitivity analysis comparing included and excluded participants on demographic characteristics and WCST outcomes. SRD could not be calculated for excluded participants because they lacked valid color or shape rule trials, which was the reason for their exclusion.
