# Supplementary material for: Relatively independent inhibitory control and cognitive flexibility deficits on the WCST in children with ADHD: age-related change patterns and cognitive profiles
Source: Front Psychiatry. 2026 Jul 29;17:1874399. doi: 10.3389/fpsyt.2026.1874399 (PMC13461803; doi:10.3389/fpsyt.2026.1874399)
Supplement: Supplementary file 1 [file Image1.pdf]

Supplementary Figure S1

Schematic diagram of the computerized Wisconsin Card Sorting Test (WCST) interface

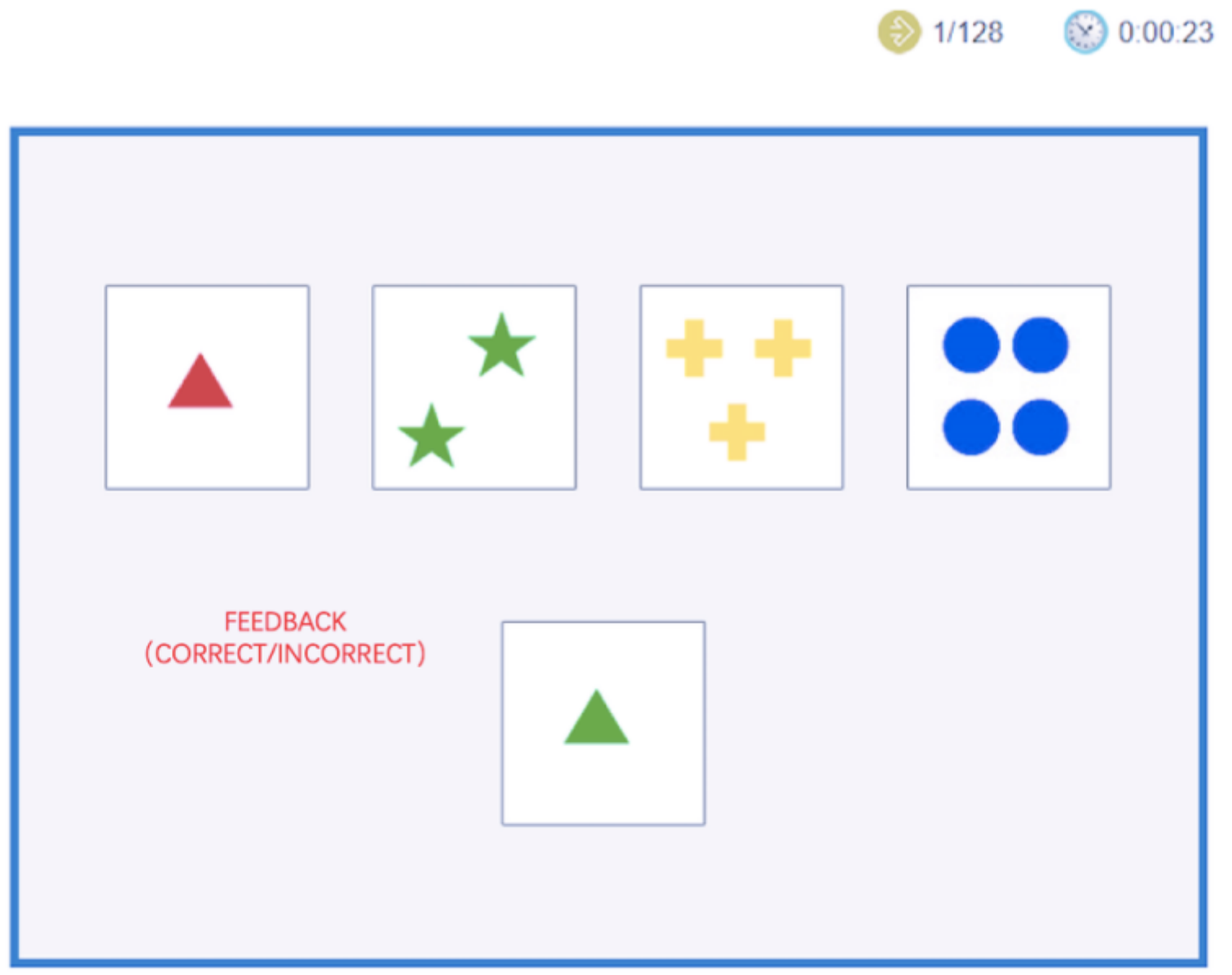

Supplementary Figure S1. Schematic diagram of the computerized Wisconsin Card Sorting Test (WCST) interface. Four stimulus cards are displayed at the top of the screen (one red triangle, two green stars, three yellow crosses, four blue circles). Response cards are presented at the bottom. After each match, the computer provides immediate feedback ("correct" or "incorrect").
