# Supplementary material for: Relatively independent inhibitory control and cognitive flexibility deficits on the WCST in children with ADHD: age-related change patterns and cognitive profiles
Source: Front Psychiatry. 2026 Jul 29;17:1874399. doi: 10.3389/fpsyt.2026.1874399 (PMC13461803; doi:10.3389/fpsyt.2026.1874399)
Supplement: Supplementary file 2 [file Table1.pdf]

Supplementary Table S1

Demographic and clinical characteristics of the ADHD sample (N = 281)

Variable	Value
Age, years (Mean ± SD)	9.8 ± 1.7
Range	7–12
Inattention subscale (Range: 0-3)	14.81 ± 5.39
Hyperactivity/Impulsivity subscale(Range: 0-3)	10.55 ± 6.07
Average item score of SNAP-IV	1.41 ± 0.58
Full-Scale IQ (M ± SD)	96.19 ± 23.78

Note: SNAP-IV = Swanson, Nolan, and Pelham Rating Scale-IV; SPM = Standard Progressive Matrices. All participants were first-visit, treatment-naïve children with ADHD
