# Supplementary material for: Relatively independent inhibitory control and cognitive flexibility deficits on the WCST in children with ADHD: age-related change patterns and cognitive profiles
Source: Front Psychiatry. 2026 Jul 29;17:1874399. doi: 10.3389/fpsyt.2026.1874399 (PMC13461803; doi:10.3389/fpsyt.2026.1874399)
Supplement: Supplementary file 3 [file Table2.pdf]

Supplementary Table S2
Gaussian mixture model comparison for two-, three-, and four-class solutions

Classes	BIC	AIC	LogLik	n per class
2	-4713.8	4684.7	-2334.3	107, 174
3	-4700.7	4657.1	-2316.5	48，86，147
4	-4709.3	4647.4	-2306.7	45，59，70，107

Note: BIC = Bayesian Information Criterion; AIC = Akaike Information Criterion. Lower BIC values indicate better model fit. The two-class solution was selected as optimal because it yielded the lowest BIC. The three-class solution produced a small intermediate subgroup (n = 48) without clear theoretical interpretation, and the four-class solution showed a higher BIC than the two-class solution, suggesting overfitting.*
