# Supplementary material for: Relatively independent inhibitory control and cognitive flexibility deficits on the WCST in children with ADHD: age-related change patterns and cognitive profiles
Source: Front Psychiatry. 2026 Jul 29;17:1874399. doi: 10.3389/fpsyt.2026.1874399 (PMC13461803; doi:10.3389/fpsyt.2026.1874399)
Supplement: Supplementary file 4 [file Table3.pdf]

Supplementary Table S3.

Sensitivity analysis comparing included and excluded participants on demographic characteristics and WCST outcomes.

**Characteristic**	**Included Participants (n = 281)**	**Excluded Participants (n = 24)**
Age (years), M±SD	9.8 ± 1.7	9.0 ± 1.7
Male, n (%)	193 (68.7%)	20 (83.3%)
Perseverative Error Rate (%), M±SD	56.8 ± 13.2	68.4 ± 9.3
Shape Rule Disadvantage (%), M±SD	12.0 ± 20.3	NA*
Overall Accuracy (%), M±SD	60.3 ± 14.2	32.8 ± 10.8

Note. SRD could not be calculated for excluded participants because they lacked valid color or shape rule trials, which was the reason for their exclusion.
